# Notch1 and Galectin-3 Modulate Cortical Reactive Astrocyte Response After Brain Injury

**DOI:** 10.3389/fcell.2021.649854

**Published:** 2021-06-16

**Authors:** Tais Novaki Ribeiro, Lina Maria Delgado-García, Marimelia A. Porcionatto

**Affiliations:** Laboratory of Molecular Neurobiology, Department of Biochemistry, Universidade Federal de São Paulo, São Paulo, Brazil

**Keywords:** neurogenic program, astrocyte reactivation, traumatic brain injury, dedifferentiation, neurogenic signaling pathway, NICD, Jagged, Hes5

## Abstract

After a brain lesion, highly specialized cortical astrocytes react, supporting the closure or replacement of the damaged tissue, but fail to regulate neural plasticity. Growing evidence indicates that repair response leads astrocytes to reprogram, acquiring a partially restricted regenerative phenotype *in vivo* and neural stem cells (NSC) hallmarks *in vitro*. However, the molecular factors involved in astrocyte reactivity, the reparative response, and their relation to adult neurogenesis are poorly understood and remain an area of intense investigation in regenerative medicine. In this context, we addressed the role of Notch1 signaling and the effect of Galectin-3 (Gal3) as underlying molecular candidates involved in cortical astrocyte response to injury. Notch signaling is part of a specific neurogenic microenvironment that maintains NSC and neural progenitors, and Gal3 has a preferential spatial distribution across the cortex and has a central role in the proliferative capacity of reactive astrocytes. We report that *in vitro* scratch-reactivated cortical astrocytes from C57Bl/6J neonatal mice present nuclear Notch1 intracellular domain (NICD1), indicating Notch1 activation. Colocalization analysis revealed a subpopulation of reactive astrocytes at the lesion border with colocalized NICD1/Jagged1 complexes compared with astrocytes located far from the border. Moreover, we found that Gal3 increased intracellularly, in contrast to its extracellular localization in non-reactive astrocytes, and NICD1/Gal3 pattern distribution shifted from diffuse to vesicular upon astrocyte reactivation. *In vitro*, Gal3^–/–^ reactive astrocytes showed abolished Notch1 signaling at the lesion core. *Notch1* receptor, its ligands (*Jagged1* and *Delta-like1*), and *Hes5* target gene were upregulated in C57Bl/6J reactive astrocytes, but not in Gal3^–/–^ reactive astrocytes. Finally, we report that Gal3^–/–^ mice submitted to a traumatic brain injury model in the somatosensory cortex presented a disrupted response characterized by the reduced number of GFAP reactive astrocytes, with smaller cell body perimeter and decreased NICD1 presence at the lesion core. These results suggest that Gal3 might be essential to the proper activation of Notch signaling, facilitating the cleavage of Notch1 and nuclear translocation of NICD1 into the nucleus of reactive cortical astrocytes. Additionally, we hypothesize that reactive astrocyte response could be dependent on Notch1/Jagged1-Hes5 signaling activation following brain injury.

## Introduction

Cortical astrocytes are highly specialized glial cells that actively participate in brain functions’ homeostasis (Verkhratsky et al., 2017). Injuries to the central nervous system (CNS) challenge astrocytes to resume tissue homeostasis by activating specific cell programs, characterizing a reactive cell-state. There are multiple functional reactive astrocyte profiles, varying depending on lesion type and severity (Verkhratsky et al., 2012; Verkhratsky and Parpura, 2016; Escartin et al., 2021). In the context of brain injury, reactive astrocytes perform a protective role. In the acute phase after brain damage, the reparative response is exclusively neuroprotective and might become both positive and negative in a chronic advanced phase. Neuroprotection includes regulating neural plasticity, facilitating the generation of neurons, axonal sprouting, and controlling the number and function of synapses (Pekny et al., 2019). *In vivo* and *in vitro* studies had shown this reparative response. Reactive astrocytes isolated from injured brains gave rise to neurospheres *in vitro* (Buffo et al., 2008; Shimada et al., 2012; Sirko et al., 2015). *In vivo*, cortical astrocytes reproduced a neurogenic response in transgenic mice with Rbpj-κ-depleted astrocytes submitted to traumatic brain injury (Zamboni et al., 2020) and reactive astrocytes activated a neurogenic program after stroke in Notch-depleted striatal astrocytes transgenic (Magnusson et al., 2020; Santopolo et al., 2020).

Thus, these complex molecular and structural changes could be targeted to promote neuroregeneration. The most recent consensus report on reactive astrocytes highlights the importance of clarifying the contribution of astrocyte-associated signaling pathways to the pathogenesis of specific neurological conditions, as well as in the development of astrocyte-guided therapies (Escartin et al., 2021). In this work, we address Notch1 signaling pathway activation and the role of Galectin-3 (Gal3) in reactive astrocytes following a traumatic injury to the brain.

Notch receptor is a transmembrane protein that signals through cell-cell interactions through its ligand Delta-like or Jagged, in mammals, which is also a transmembrane protein on a neighboring cell. Ligand binding promotes two proteolytic cleavage events in the Notch receptor, releasing the Notch intracellular domain (NICD), which then translocates to the nucleus and cooperates with the DNA-binding protein Rbpj and its co-activator Mastermind-like (MAML) to promote target genes transcription (Hes1, Hes5, Hey). The Notch signaling pathway outcome depends on the cellular context and can result in proliferation, differentiation, and apoptosis. Notch is a critical element of cell fate decision during neurodevelopment, specifying radial glial cell identity (Gaiano et al., 2000; Gaiano and Fishell, 2002) and promoting differentiation of astrocytes, in detriment of oligodendrocytes and neurons (Grandbarbe et al., 2003). Notch has been extensively studied in neurogenesis in the adult brain, as it has a pivotal role in maintaining neural stem cell pool in the neurogenic niches (Ables et al., 2010; Engler et al., 2018). Notch is also implicated in neuropathological contexts, including astrocyte proliferative response to brain injury (Shimada et al., 2011; LeComte et al., 2015; Zhong et al., 2018; Santopolo et al., 2020) and regulation of reactive astrocyte morphology and response upon inflammatory stimuli (Acaz-Fonseca et al., 2019).

Gal3 is a lectin that binds to galactose residues in glycoproteins and glycolipids and oligomerizes through its N-terminal domain. Gal3 oligomerization generates a dynamic and complex structure capable of regulating diffusion, compartmentalization, and endocytosis of glycoproteins and membrane glycolipids. Gal3 contributes to cell-cell and cell-matrix interactions when located at the extracellular matrix, but it is also found in the nucleus and cytoplasm. The ubiquitous Gal3 distribution explains its broad influence in cellular functions such as apoptosis, proliferation, migration, angiogenesis, RNA splicing, and surface to nuclear signal transport (Nieminen et al., 2007; Nabi et al., 2015). Gal3 is expressed at different levels throughout the CNS and was shown to have a widespread expression profile in the cerebral cortex (John and Mishra, 2016). Overexpression of Gal3 was correlated with a reactive cell-state and reactive astrocyte ability to re-create neural stem cell properties *in vitro* (Sirko et al., 2015). Furthermore, Gal3 plays a significant role in neuroinflammation (Yip et al., 2017; Srejovic et al., 2020) and cancer stemness maintenance (Newlaczyl and Yu, 2011; Nangia-Makker et al., 2018).

Acknowledging the extraordinary complexity of astrocyte response to traumatic brain injury, we hypothesized that the interaction of Gal3 and Notch1 in reactive astrocytes is critical for the maintenance and proper function of the adult brain. Here we report that Gal3 modulates Notch1 signaling pathway in reactive astrocytes, and we provide new evidence of the participation of Notch1-Hes5 signaling axis activation in reactive astrocytes.

## Materials and Methods

### Animals

The animals were handled following National Institute of Health (NIH) regulations, and all procedures were approved by the Committee on Ethics in the Use of Animals from Universidade Federal de São Paulo (CEUA n. 7740290318; CEUA n. 2451111116). CEDEME/UNIFESP Animal Facility supplied isogenic C57Bl/6J and Gal3 knockout (Gal3^–/–^) mice (Supplementary Figure 1) aged 6 and 45 days. Gal3 knockout (Gal3^–/–^) mice were generated by Fu-Tong Liu group (Hsu et al., 2000) and were obtained from Biotério Central, Faculdade de Medicina, USP (Rede PREMIUM^1^). The animals were housed in standard cages, maintained under controlled light-dark cycles (12/12 h; lights on at 7 a.m.) with food and water available *ad libitum*. We made all efforts to minimize suffering and the number of animals used.

### Primary Astrocyte Culture and *in vitro* Model of Astrocyte Reactivity

Astrocyte isolation from mice cortices was adapted from Yang et al. (2009). The animals were anesthetized with intraperitoneal injection of 2% xylazine (10 mg/kg, Syntec, Barueri, SP, Brazil) and 10% ketamine hydrochloride (100 mg/kg, Syntec, Barueri, SP, Brazil) and euthanized by decapitation. The brain was removed from the skull and the cortices dissected and placed in Hanks/DNAse solution. The tissue was mechanically dissociated and incubated with 0.25% trypsin (Sigma Aldrich Corporation, Saint Louis, EUA) in Versene/DNAse solution for 20 min. Trypsin activity was blocked with fetal bovine serum (FBS, Cultilab, Campinas, SP, Brazil), and cells were homogenized and dissociated in Versene/DNAse solution. The cells were suspended in DMEM/F12 medium containing 100 U/mL penicillin/streptomycin (Gibco, Grand Island, EUA), 200 mM L-glutamine (Sigma Aldrich Corporation, Saint Louis, EUA), and 10% FBS, filtered through a 40 μm cell strainer and plated in T25 flasks coated with poly-l-lysine. Half-medium changes were performed every 2 days.

*In vitro* model of astrocyte reactivity was adapted from Yang et al. (2009). Astrocytes at first passage were seeded in 13 mm coverslips for microscopy analysis and 60 mm dishes for RNA extraction and flow cytometry assay. After reaching 80–90% confluency, the astrocyte monolayer was scratched with a 10 μm pipette tip. The scratch pattern for coverslips was a “cross” composed of one horizontal and one vertical scratch, and for 60 mm dishes, the pattern was a “grid” composed of several scratches 0.5 cm distant from each other. The scratch assay causes cell detachment and loss of cell-cell contact, comparable to the borders of a traumatic brain injury. Three days post lesion (3dpl), cultures were highly enriched with reactive astrocytes and were processed for immunocytochemistry, total RNA extraction for quantitative PCR (qPCR) and flow cytometry.

### Traumatic Brain Injury Model

Traumatic brain injury (TBI) model was previously described in Mundim et al. (2019). Briefly, adult 45-days-old C57BL/6J (*n* = 3) and Gal3^–/–^ (*n* = 3) mice were anesthetized with intraperitoneal injection of 0.2% acepromazine (2.5 mg/kg, Vetnil, Louveira, SP, Brazil), 2% xylazine (20 mg/kg), 10% ketamine hydrochloride (80 mg/kg), and 0.05% fentanyl (0.5 mg/kg, Syntec, Brazil) and were placed in a stereotaxic frame. Mice were submitted to a unilateral penetrating lesion performed with a 22-gauge needle (0.7 mm) in the somatosensorial cortex (stereotaxic coordinates from bregma: AP + 0 mm; ML + 1 mm; DV − 0.7 mm) three times, for 2 min. Three days later (3dpl), mice were deeply anesthetized with intraperitoneal injection of 2% xylazine, 10% ketamine hydrochloride, and 0.05% fentanyl and intracardially perfused with 4% paraformaldehyde (PFA). Brains were removed from the skull, postfixed in 4% PFA overnight at 4°C, submersed in 30% sucrose at 4°C, and frozen using dry ice. Cryostat coronal sections (40 μm) were collected and prepared for immunohistochemistry.

### Immunocytochemistry and Immunohistochemistry Analysis

For immunocytochemistry assays, cells were previously seeded on coverslips and submitted to a model of *in vitro* astrocyte reactivity; alternatively, cells were maintained under the same medium conditions as control. After 3dpl, both control and reactive astrocytes were fixed in 4% PFA and permeabilized with PBS-0.1% Triton (PBST) for 5 min. After sequential washes with PBS 1×, the cells were incubated at room temperature for 1 h in blocking solution (5% bovine serum albumin in PBST). Primary antibodies were diluted in blocking solution and incubated overnight at 4°C. The cells were washed with PBS 1× and incubated at room temperature for 2 h with the corresponding secondary antibodies and fluorescence nuclear counterstain DAPI. Primary antibodies: chicken anti-Glial fibrillary acidic protein, GFAP (AB5541, 1:1,000, Millipore, Massachusetts, United States); rat anti-Gal3 (sc-23938, 1:250, Santa Cruz, Texas, United States); rabbit anti-Jagged1 (orb10065, 1:500, Biorbyt Ltd., Cambridge, United Kingdom); mouse anti-Notch1 (ab128076, 1:500, Abcam, Cambridge, United States). It is important to note that the anti-Notch1 antibody ab128076 strongly recognizes the activated form of the intracellular domain of Notch1 protein (NICD1). The unprocessed Notch1 protein is recognized with lower affinity. Secondary antibodies: Alexa Fluor 488-conjugated goat anti-chicken IgG; Alexa Fluor 488-conjugated goat anti-rabbit IgG; Alexa Fluor 594-conjugated goat anti-mouse IgG; Alexa Fluor 647- conjugated goat anti-rat IgG; Alexa Fluor 647-conjugated goat anti-chicken IgG (1:500, Invitrogen, Massachusetts, United States). Coverslips were mounted onto slides with Fluoromount G solution (Electron Microscopy Sciences, Hatfield, Pennsylvania, United States).

For immunohistochemistry assays, after several washes in PBST, the sections were pre-incubated for 1 h at room temperature in 5% normal goat serum. Immunohistochemistry was performed by incubating the sections overnight (2–8°C) with selected primary antibodies. Next day, sections were rinsed in 0.1% PBST and incubated for 90–120 min with the corresponding secondary antibody. Finally, the sections were rinsed and mounted onto slides with Fluoromount G. Primary antibodies: chicken anti-GFAP (1:500), mouse anti-Notch1 (1:500). Secondary antibodies: Alexa Fluor 488-conjugated goat anti-mouse IgG and Alexa Fluor 647-conjugated goat anti-chicken IgG (1:500).

The immunofluorescence was analyzed by confocal microscopy using Leica TCS SP8 microscope (Houston, United States) and Zeiss Observer Z.1. (Jena, Germany) and images were processed using ImageJ software (1.49v^2^).

### Vesicular Assay and ROI Analyses

GFAP and Nuclear NICD1 Normalized Fluorescence: For GFAP protein expression in selected areas, first we used the “histogram” and “threshold” tools to establish a minimum intensity value and determine regions of interest (ROI). Next, we measured the mean gray value (GV) and integrated density (IntD) and selected additional ROI to measure the background fluorescence (bk). GV was corrected through the subtraction of the mean bk. GV and IntD were used to compare GFAP fluorescence intensity between groups. For nuclear NICD1 analysis, single cell analysis of protein expression was calculated using normalized fluorescence. First, ROI were drawn around cells and were determined: area (A), mean gray value (GV), and the integrated intensity (IntD). Next, background (bk) fluorescence was measured by drawing a ROI in an area around the cells or nucleus of interest and determined a mean gray value of the background fluorescence (GVbk). Normalized fluorescence was calculated as A-(IntD x GVbk) and used to compare NICD1 nuclear fluorescence intensity between groups. NICD1/Jagged1 colocalization: To determine NICD1/Jagged1 colocalization, ROIs were drawn around cells according to GFAP immunostaining and the “colocalization threshold” tool was used. Vesicular assay: The “threshold” and “create selection” tool and “ROI manager, -AND- option” were used to detect and count the number of vesicles in the nucleus and in the intracellular compartment. It was established a minimum intensity value through the “histogram” tool in each channel to configure a “threshold” and a vesicular size of 0.03 μm (± 0.02 SD) to create ROIs. Cell body perimeter (morphological analysis): The “Simple Neurite Tracer (SNT)” plugin and “perimeter” tool of the convex hull area were used for the reconstruction of each cell and the morphological analysis. GFAP/Notch1 distribution: Lesioned areas were localized by GFAP staining and the perimeters of the lesion were defined using the “enlarge” tool, with a distance of 50 μm between each concentric grid.

### Quantitative RT-PCR Analysis

We evaluated mRNA expression profile of Notch signaling pathway members (*Notch1*, *Jagged1*, *Delta-like1, Hes1, Hes5, Mash1*) in control and reactive astrocyte cultures of C57BL/6J and Gal3^–/–^ mice. RNA was extracted according to manufacturer’s recommendations using PureLink RNA Micro Kit (cat n. 12183-016, Invitrogen, MA, United States). RNA was quantified and quality was assessed using spectrophotometer NanoVue Plus (GE Healthcare, Buckinghamshire, United Kingdom). Total RNA was reverse transcribed using High Capacity cDNA Reverse Transcription Kit (cat n. 4368814, Applied Biosystems, MA, United States). Quantitative RT-PCR (qPCR) was performed using Fast SYBR Green Master Mix (cat n. 4385610, Applied Biosystems) in an Applied Biosystems 7500 Real-Time PCR System. Thermal cycling conditions were 95°C for 20 s, 40 × 95°C for 3 s, and 58°C for 30 s. The dissociation curve was performed at 95°C for 1 min, 60°C for 30 s, and 95°C for 30 s. The primers used were *Dll1*–sense 5′-CCCATCCGATTCCCCTTCG-3′ and antisense 5′-GGTTTTCTGTTGCGAGGTCATC-3′; *Gapdh*– sense 5′-AGGTCGGTGTGAACGGATTTG-3′ and antisense 5′- TGTAGACCATGTAGTTGAGGTCA-3′; *Hes1*–sense 5′-CTAT CATGGAGAAGAGGCGAAG-3′ and antisense 5′-CCGGGAG CTATCTTTCTTAAGTG-3′; *Hes5*–sense 5′-CCAAGGAGAAA AACCGACTG-3′ and antisense 5′-TCCAGGATGTCGGCCTTC TC-3′; *Jag1*–sense 5′-GATTGCCCACTTCGAGTATCA-3′ and antisense 5′-CGTTCTGGTCACAGGCATAA-3′; *Mash1*–sense 5′-CTTGAACTCTATGGCGGGTT-3′ and antisense 5′-TAAAG TCCAGCAGCTCTTGTT-3′; and *Notch1*–sense 5′-CCCGCTG TGAGATTGATGTTA-3′ and antisense 5′-CACCTTCATAAC CTGGCATACA-3′. Three biological replicates for each group and three technical replicates for each gene were analyzed. Gene expression was normalized to *Gapdh* expression and the 2^ΔΔCT^ method (Livak and Schmittgen, 2001) was used for relative quantification analysis.

### Flow Cytometry Analysis

Cells were detached using a 0.25% trypsin and suspended in astrocyte medium. Cell membranes in the pellet were stabilized by shaking for 1 h. For the extracellular analysis, the cells were suspended in blocking buffer (2% FBS in PBS) for 20 min and incubated with the primary antibody rat anti-Gal3 (1:100) for 1 h under constant agitation. The cells were washed and incubated with the secondary antibody, Alexa Fluor 488- conjugated goat anti-rat IgG (1:500) under the same conditions. Finally, cells were fixed in 4% PFA for 20 min, washed, and the pellet was suspended in PBS. Conversely, for intracellular analyses, after membrane stabilization, cells were fixed in 4% PFA for 20 min and permeabilized using a Perm Wash Buffer solution for 1 h (BD Bioscience, San Jose, United States). Concomitantly, the pellet was incubated with the corresponding primary antibody, rat anti-Gal3 (1:100) and/or chicken anti-GFAP (1:1,000), depending on the experiment, for 1 h under constant agitation. Secondary antibodies used were Alexa Fluor 488-conjugated goat anti-rat IgG and Alexa Fluor°647-conjugated goat anti-chicken IgG (1:500). For both experiments, one aliquot of unstained control cells (negative control) was used for evaluating autofluorescence (omission of antibodies) and other to assess the non-specific binding of the secondary antibody (omission of the primary antibody). Non-scratched astrocytes were used as control. Finally, the samples were suspended in PBS 1X and analyzed in a FACS Canto II flow cytometer (BD Biosciences, Mountain View, CA, United States). A minimum of 10.000 events per sample were collected, and data were analyzed using Cyflogic^TM^ software (CyFlo Ltd., Finland).

### Statistical Analysis

Statistical analysis of data and graphical representations were performed using GraphPad Prism (version 5.0)^3^ and Microsoft Excel (version 2016)^4^. The graphs presented show mean ± standard error. The difference among groups was assessed using unpaired Student’s *t*-test unless stated otherwise. The results were reported in absolute and relative values and the level of statistical significance adopted was 5% (*p* < 0.05). Differences among groups are indicated in the graphs with asterisks: ^∗^*p* ≤ 0.05, ^∗∗^*p* ≤ 0.01, ^∗∗∗^*p* ≤ 0.001.

## Results

### Notch1 Signaling Is Activated in Reactive Astrocytes *in vitro*

**FIGURE 1:**
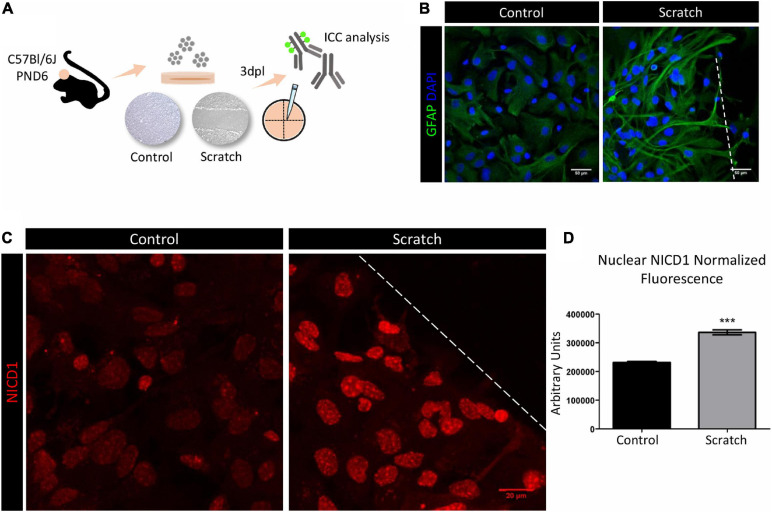
Notch1 signaling is activated in reactive astrocytes *in vitro*. **(A)** Experimental design: astrocytes were isolated from the cortex of postnatal day six (PND6) C57BL/6J mice and cultured until confluency in 13 mm coverslips. Astrocyte reactivity was induced by scratch assay, and expression of the reactivity marker GFAP was analyzed 3 days post lesion (3dpl) by immunocytochemistry and confocal imaging. **(B)** Scratch-induced astrocytes are GFAP^+^ and show reactive morphology. Scale bar: 50 μm. **(C)** Representative confocal images of NICD1 staining in control and reactive astrocytes. Dashed line indicates the scratch border. Scale bar: 20 μm. **(D)** Normalized fluorescence analysis of nuclear NICD1 immunostaining revealed increased NICD1 nuclear localization in reactive astrocytes compared to control (***p ≤ 0.001); unpaired Student’s *t*-test, *n* = 154 nuclei in scratch / 217 nuclei in control, three culture replicates. Data are mean ± SEM. Nuclei were stained with 4’,6-diamidino-2-phenylindole (DAPI; blue).

To investigate the Notch signaling role in astrocyte reactivation, we initially established a scratch-assay model to promote astrocyte reactivity *in vitro*. Confluent astrocyte cultures were scratch-activated, and their activation status was analyzed 3 days post-lesion (3 dpl, Figure 1A). Under control conditions, astrocytes showed a polygonal to fusiform and flat morphology, and when reactivated by scratching, we observed morphological changes that included hypertrophic cell bodies and increased secondary processes emerging from primary branches. These processes frequently overlapped in a projection to the lesion core and showed increased expression of GFAP, shown by immunocytochemical analysis (Figure 1B). We next evaluated the activation of Notch signaling in primary cultures of reactive astrocytes from C57Bl/6J mice. Using an anti-Notch1 that mainly recognizes NICD1, we observed stronger immunolabeling in the nucleus of reactive astrocytes when compared to control astrocytes (Figures 1C,D), as well as in cytoplasmic vesicles (Supplementary Figure 2). Even though the antibody we used in this work does not specifically recognize NICD1 generated by γ-secretase cleavage between positions Gly1743 and Val1744 of Notch, our results show nuclear localization of NICD1, suggesting that the Notch1 signaling pathway is activated in reactive astrocytes.

To investigate the molecular mechanisms underlying astrocytes response, we next quantitatively compared the presence of NICD1 and Notch1 receptor ligand, Jagged1, in scratch-reactivated astrocytes in two distinct regions: (i) astrocytes at the lesion core and (ii) astrocytes located away from the lesion (periphery). We observed that reactive astrocytes located at the lesion border showed more NICD1 and Jagged1, with a strong colocalization pattern in the lesion core compared to the periphery (Supplementary Figure 3). This result suggests that Notch1 signaling is activated in the astrocytes closer to the injury site, where cell-cell interaction was disrupted by scratching the cell monolayer.

### *In vitro* Astrocyte Activation Increases Intracellular Gal3

**FIGURE 2:**
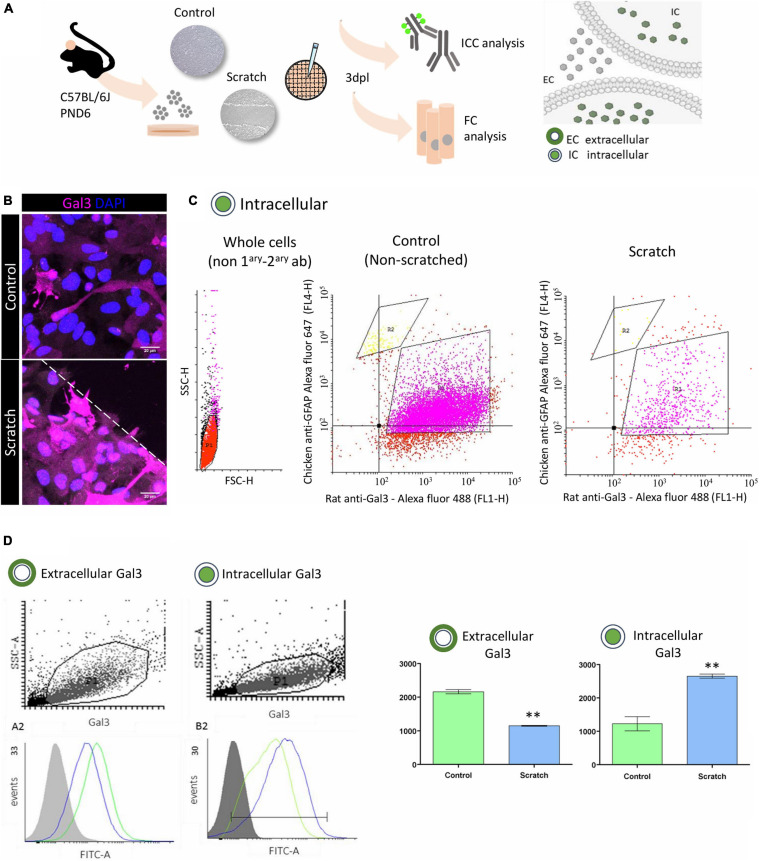
*In vitro* astrocyte activation increases intracellular Gal3. **(A)** Experimental design: Gal3 localization in control (non-scratched) and reactive (scratched) astrocytes was analyzed by immunocytochemistry and flow cytometry. **(B)** Representative Z-stack confocal images of Gal3 immunolocalization in control and reactive astrocytes *in vitro*. Dashed line indicates scratch border. Scale bar: 20 μm. **(C)** Flow cytometry cell distribution analysis of control and reactive astrocytes showed two subpopulations: R1, GFAP^+^/Gal3^+^ (75.70% of total number of cells) and R2, GFAP^+^/Gal3^–^ (3.25% of total number of cells). Data correspond to geometric mean. **(D)** Analysis of Gal3 distribution between the extracellular and intracellular compartments in control and reactive astrocytes showed that at 3dpl, control astrocytes presented more extracellular Gal3 compared to reactive astrocytes (**p ≤ 0.01, unpaired Student’s *t*-test, *n* = 3). Conversely, intracellular Gal3 was increased in reactive astrocytes compared to control (**p ≤ 0.01, unpaired Student’s *t*-test, *n* = 3). Data correspond to mean ± SD. A minimum of 10,000 events per sample per experiment were analyzed, and data was processed using Cyflogic^TM^ software (CyFlo Ltd., Finland).

Gal3 is ubiquitously distributed in the cerebral cortex, and it has been related to the modulation of diverse intra- and extracellular processes. We observed strong Gal3 immunostaining in reactive astrocytes and sought to investigate the cellular location of Gal3 by using conventional flow cytometry analysis to search for its presence at the cell membrane (non-permeabilized) and in intracellular (permeabilized) compartments (Figures 2A,B).

First, we characterized the population of control (non-reactive) and reactive astrocytes cultures depending on the intracellular content of GFAP and Gal3 protein. Scatter plot analysis of both control and reactive astrocytes showed two subpopulations: GFAP^+^/Gal3^+^ cells corresponding to the R1 gate (75.70%) and a second population, GFAP^+^/Gal− cells corresponding to the R2 gate (3.25%), with no statistical differences between control and reactive astrocytes in R1 and R2 gates (Figure 2C). Next, in a second experiment, and given that GFAP^+^/Gal3^+^ (R1 gate) corresponded to 75.70% of the cell population, we sought to evaluate the location of Gal3 protein between the extracellular (non-permeabilized) and intracellular (permeabilized) compartments of control and reactive astrocytes. The Gal3 histogram of the non-permeabilized assay showed a higher amount of the protein at the surface of control (non-reactive) astrocytes (2,163 ± 62.69) when compared with reactive astrocytes (1,151 ± 13.04) (Figure 2D). Conversely, the histogram of the permeabilized assay showed that Gal3 was predominantly present in the intracellular compartment of reactive astrocytes (2,655 ± 60.58) when compared to control (non-reactive) astrocytes (1,229 ± 211.7). Altogether, these results suggest that there were two main populations of GFAP^+^ cells (R1 and R2 gates) and that at 3dpl, Gal3 is preferentially localized in the intracellular compartment of reactive astrocytes.

### NICD1 and Gal3 Colocalize in Vesicles in Reactive Astrocytes

**FIGURE 3:**
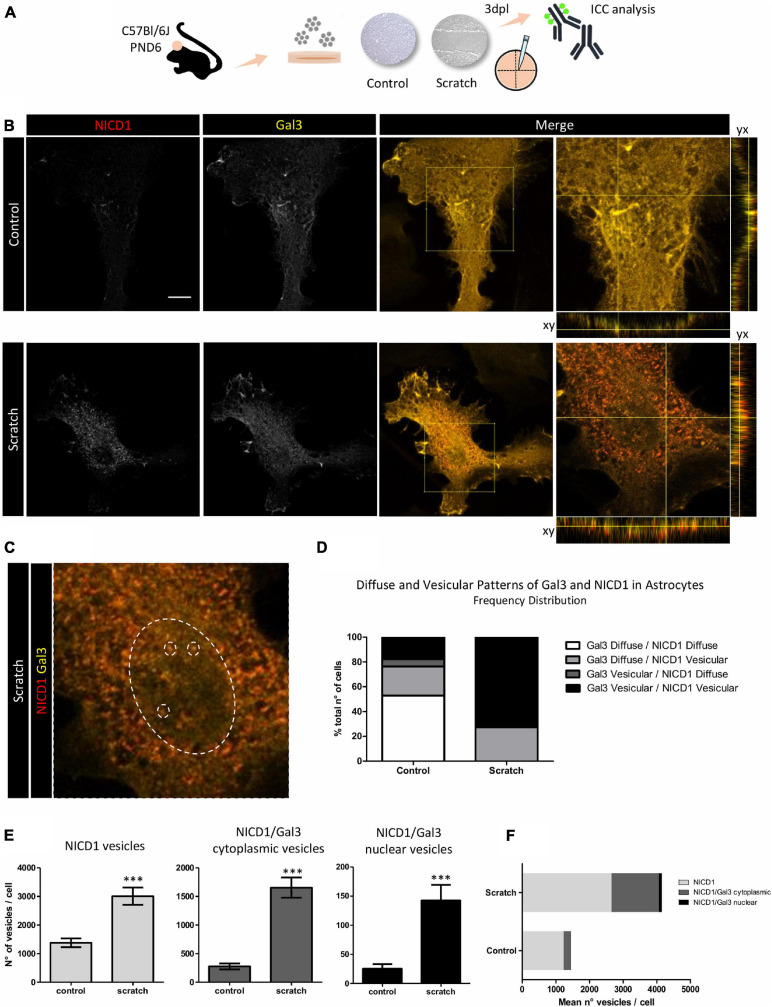
NICD1 and Gal3 colocalize in vesicles in reactive astrocytes. **(A)** Experimental design: NICD1 and Gal3 were immunolabeled in scratch-reactivated and control (non-reactivated) astrocytes and analyzed by confocal microscopy. **(B,left)** Representative confocal Z-stack gray scale images of NICD1 and Gal3 in control and reactive astrocytes. **(B,right)** Merged images of NICD1 and Gal3 and detailed images with the orthogonal view of the immunostaining. Scale bar: 20 μm. **(C)** Zoom image from one reactive astrocyte: large dashed circle indicates the nucleus and the three small dashed circles inside the large circle indicate representative vesicles, highlighting NICD1 and Gal3 intranuclear colocalization. **(D)** Frequency analysis of NICD1 and Gal3 immunolabeling patterns (diffuse and vesicular) in control and reactive astrocytes. Values are plotted as percentage of total number of cells analyzed: 52.9% control astrocytes showed Gal3/NICD1 diffuse pattern; 23.5% Gal3 diffuse/NICD1 vesicular; 5.9% Gal3 vesicular/NICD1 diffuse, and 17.6% Gal3 vesicular/NICD1 vesicular. Reactive astrocytes showed 27.3% Gal3 diffuse/NICD1 vesicular pattern and 72.7% Gal3 vesicular/NICD1 vesicular (control *n* = 17 cells, scratch *n* = 22 cells). **(E)** Quantification analysis of NICD1^+^ and NICD1^+^Gal3^+^ vesicles revealed a higher number of NICD1^+^ vesicles, NICD1^+^Gal3^+^ cytoplasmic vesicles and NICD1^+^Gal3^+^ nuclear vesicles in reactive astrocytes compared to control. (***p ≤ 0.001; unpaired Student’s *t*-test; control *n* = 18 cells, scratch *n* = 27 cells). **(F)** Graphical representation of mean number of NICD1^+^, NICD1^+^Gal3^+^ cytoplasmic and nuclear vesicles per cell in control and reactive astrocytes. (control *n* = 18 cells, scratch *n* = 27 cells).

Since we showed NICD1 nuclear localization in reactive astrocytes and Gal3 is distributed intracellularly, we asked whether NICD1 and Gal3 colocalized in reactive astrocytes. Initially, we analyzed and found two distinct Gal3 immunostaining patterns: diffuse and vesicular. We were able to distinguish granules with intense staining for the vesicular pattern, and in the diffuse pattern, we observed a weaker Gal3 signal. Similarly, the distribution patterns could also be attributed to NICD1 (Figures 3A–C and Supplementary Figure 4). Of note, Gal3 was predominantly distributed in a diffuse pattern in non-reactive astrocytes (76.5% of astrocytes, Figure 3D), and in contrast, 72.7% of reactive astrocytes presented vesicular Gal3. Interestingly, NICD1 distribution pattern shift was more pronounced in control astrocytes, in which NICD1 was distributed in vesicular (41.2%) and diffuse (58.8%) patterns. However, in 100% of reactive astrocytes analyzed, NICD1 was found in vesicular pattern (Figure 3D). When classifying astrocytes according to both NICD1 and Gal3 distribution patterns, we noticed that 52.9% of control astrocytes had diffuse Gal3 and NICD1, while none of the reactive astrocytes presented the same pattern (Figure 3D). In contrast, Gal3 vesicular/NICD1 vesicular pattern was found in 72.7% of the reactive astrocytes. It is important to note that both Gal3 and NICD1 could be mostly found in a diffuse pattern in control astrocytes. Upon lesion and astrocyte reactivation, the vesicular pattern became prevalent for both NICD1 and Gal3. This observation supports the hypothesis that NICD1 and Gal3 interact in reactive astrocytes.

To address this hypothesis, we analyzed if Gal3 and NICD1 vesicles colocalized in reactive astrocytes. In line with the previous observation on the distribution pattern of NICD1, we showed that reactive astrocytes have more NICD1^+^ vesicles than control astrocytes (Figure 3E). The colocalization analysis revealed that there are more NICD1^+^/Gal3^+^ vesicles in reactive astrocytes, both in the cytoplasm and nucleus (Figure 3E). By quantifying the mean number of vesicles per cell, we observed that the higher number of vesicles in reactive astrocytes were both from NICD1 vesicles and NICD1/Gal3 vesicles (Figure 3F). Also, there was a 7.12-fold increase of NICD1/Gal3 cytoplasmic vesicles in contrast to 2.15-fold increase of NICD vesicles in reactive astrocytes when compared to control astrocytes (Figure 3F).

### Notch1 Signaling Is Impaired in Gal3^–/–^ Astrocytes

**FIGURE 4:**
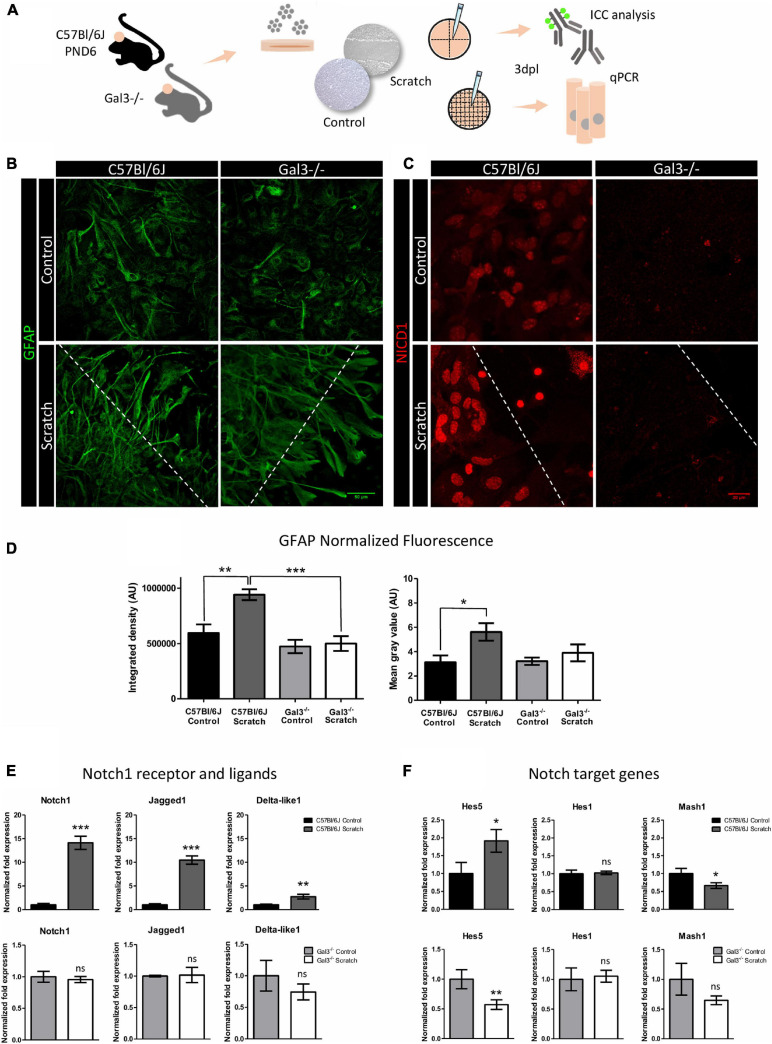
Notch1 signaling is impaired in Gal3^–/–^ astrocytes. **(A)** Experimental design: C57Bl/6J and Gal3^–/–^ astrocytes were scratch-reactivated. Protein and gene expression were analyzed by immunocytochemistry and qPCR. **(B)** Representative Z-stack confocal images of GFAP staining in C57Bl/6J and Gal3^–/–^ reactive and non-reactive astrocytes. C57Bl/6J and Gal3^–/–^ reactive astrocytes project cellular processes to the lesion border. Scale bars: 50 μm. **(C)** Gal3^–/–^ astrocytes do not activate Notch1 signaling, as shown by total absence of nuclear NICD1 immunostaining. In contrast, C57Bl/6J reactive astrocytes display strong nuclear NICD1 immunostaining, suggesting activation of Notch1 signaling. Dashed lines indicate scratch border. Scale bar: 20 μm. **(D)** GFAP normalized fluorescence analysis of C57Bl/6J and Gal3^–/–^ control and scratched-astrocytes. Values are reported in integrated density **(D,left)** and mean gray value **(D,right)**. AU = arbitrary units (***p ≤ 0.001; **p ≤ 0.01; *p ≤ 0.05). qPCR analysis of **(E)** *Notch1* receptor and ligands and **(F)** mRNA expression levels of Notch1 target genes in C57Bl/6J and Gal3^–/–^ astrocytes. Values are relative to control group and are expressed in fold change. mRNA expression level was normalized to *Gapdh*. (***p ≤ 0.001; **p ≤ 0.01; ns = not significant; unpaired Student’s *t*-test; *n* = 3 biological and technical replicates).

Since our results showed colocalization of NICD1 and Gal3 in reactive astrocytes, we asked whether Notch1 signaling would be activated upon scratch-induced reactivation of astrocytes lacking Gal3. We evaluated astrocyte reactivation status by GFAP expression and investigated the presence of NICD1 in primary cultures of astrocytes obtained from Gal3 knockout mice (Figure 4A). We noted that Gal3***^–^***^/^***^–^*** astrocytes acquired a reactive morphology upon scratch-reactivation *in vitro*; however, there were no significant changes in GFAP expression (Figures 4B,D). GFAP upregulation is one of the hallmarks of astrocyte reactivation, and our results showed this response only in C57Bl/6J reactive astrocytes (Figures 4B,D). Next, NICD1 immunostaining analysis revealed the total absence of nuclear NICD1 in Gal3***^–^***^/^***^–^*** control and scratch-reactivated astrocytes at 3dpl. In contrast, wild type (C57BL/6J) astrocytes immunomarked for nuclear NICD1 (Figure 4C). These results suggested that the absence of Gal3 impairs GFAP overexpression, therefore altering astrocyte reactive response, and impairs activation of Notch1 receptor and NICD1 translocation to the nucleus (Figures 4B–D).

Next, we asked if the lack of activation was due to a lack of Notch1 expression or a dysfunction in the signaling pathway. Further analysis of *Notch1* mRNA expression revealed that in C57BL/6J astrocytes there was a 14.1 ± 2.4 fold increase in *Notch1* expression after astrocyte reactivation (Figure 4E). In agreement with NICD1 immunostaining, *Notch1* mRNA expression did not change upon scratch-induced activation of Gal3***^–^***^/^***^–^*** astrocytes (Figure 4E). We next asked whether the mRNA expression level of Notch1 ligands, Delta-like1 and Jagged1, would be altered in C57BL/6J or Gal3***^–^***^/^***^–^*** astrocytes upon scratch-induced activation. In reactive C57BL/6J astrocytes, *Jagged1* expression increased by 10.5 ± 0.9-fold (Figure 4E), whereas the expression of *Delta-like1* increased by 2.7 ± 0.5-fold (Figure 4E). However, there were no significant changes in the expression of both ligands between Gal3***^–^***^/^***^–^*** control and reactive astrocytes (Figure 4E). To address gene expression regulation of Notch1 effectors, we analyzed *Hes5*, *Hes1*, and *Mash1* mRNA expression. Scratch-stimulus positively regulated *Hes5* transcription by 1.9 ± 0.3-fold change and negatively regulated *Mash1* (0.7 ± 0.1-fold change) in C57Bl/6J reactive astrocytes. *Hes1* mRNA expression remained unchanged (Figure 4F). In Gal3***^–^***^/^***^–^*** astrocytes, reactivation stimulus did not modify *Hes1* and *Mash1* mRNA expression level, but downregulated Hes5 expression by 0.6 ± 0.1 fold change (Figure 4F).

Collectively, these findings indicate that both ligands Jagged1 and Delta-like might promote Notch1 activation and consequent Hes5 expression in C57BL/6J reactive astrocytes. Moreover, our results indicate that Gal3 ablation impairs Notch1 signaling activation in reactive astrocytes *in vitro*, which was corroborated by mRNA expression of Notch1 target genes.

### Gal3^–/–^ Reactive Astrocytes Show Incomplete Response and Dysfunctional Notch1 Signaling Activation Following Traumatic Brain Injury

**FIGURE 5:**
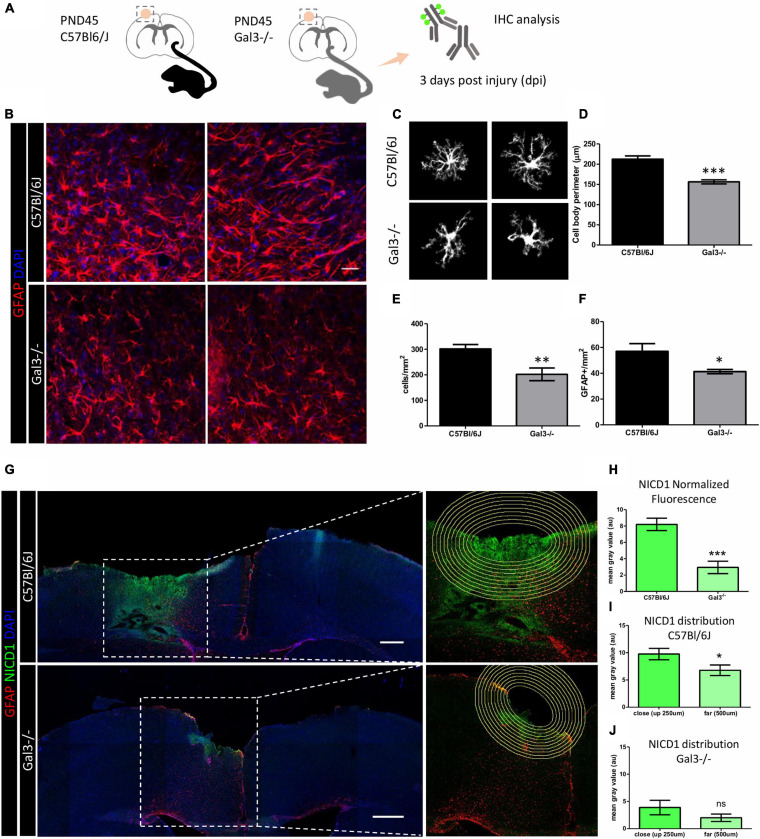
Gal3^–/–^ mice reactive astrocytes show incomplete response and dysfunctional Notch1 signaling activation following TBI. **(A)** Experimental design: Adult mice were submitted to a penetrating stab wound injury at the somatosensorial cortex and tissue sections were processed 3dpl for GFAP and NICD1 immunolocalization. **(B)** Representative confocal images of GFAP staining at TBI core. Scale bar: 50 μm. C57BL/6J reactive astrocytes extend primary branches to the lesion core and acquire a bipolar morphology. In contrast, Gal3^–/–^ reactive astrocytes do not polarize to the lesion core. **(C,D)** Gal3^–/–^ reactive astrocytes show less complex morphology and smaller cell bodies compared to C57BL/6J reactive astrocytes (***p ≤ 0.0001, unpaired *t*-test, *n* = 20 cells C57BL/6J / 20 Gal3^–/–^ cells). Quantification of the number of cells around the lesion core revealed **(E)** fewer cells per mm^2^ (**p ≤ 0.01, unpaired *t*-test, *n* = 11 C57BL/6J cells / 10 Gal3^–/–^ cells) and **(F)** fewer GFAP^+^ cells per mm^2^ (*p ≤ 0.05, unpaired *t*-test, *n* = 11 C57BL/6J cells / 10 Gal3^–/–^ cells). **(G,left)** Mosaic composition of TBI confocal images showing NICD1 distribution at the lesion core. Scale bar: 500 μm. **(G,right)** Zoom image of the section. Concentric circles with 50 μm distance between each one were drawn around the lesion core and used to quantify NICD1 labeling intensity **(H–J)**. **(H)** NICD1 immunolabeling is more intense up to 500 μm from the lesion border in C57BL/6J mice than in Gal3^–/–^ mice (***p ≤ 0.001, unpaired *t*-test). **(I)** In C57BL/6J mice, NICD1 fluorescence intensity is stronger in cells closer to the lesion border (up to 250 μm) compared to the cells that are farther away from the border of the lesion (250 μm to 500 μm) (*p ≤ 0.05, unpaired *t*-test). **(J)** NICD1 close/far distribution is not seen in Gal3^–/–^ mice (ns = not significant).

We gathered evidence of Gal3 modulatory role on Notch1 signaling by investigation of the signaling dynamics of Notch1 in scratch-reactivated astrocytes. To confirm these findings and considering that the reactive response is a progressive and active process that follows brain injury, we aimed to study astrocyte reactivity and the participation of Gal3 in C57BL/6 and Gal3***^–^***^/^***^–^*** mice submitted to a model of TBI (Figure 5A).

A general evaluation of GFAP reactive astrocytes in C57BL/6J mice showed that cells were distributed, forming net-like structures in the edge of the lesion; however, in Gal3***^–^***^/^***^–^*** mice, reactive astrocytes did not display these arrangements. Quantification of the total number of cells, the proportion of GFAP cells, and cell body perimeter as indirect parameters of reactivity response showed that Gal3***^–^***^/^***^–^*** mice had an incomplete reactive astrocyte response compared with C57BL/6J mice (Figures 5B–F).

Notch1 protein analysis showed that activation of the signaling pathway in the TBI model had a close/far dependent distribution, being higher in areas close to the lesion core and sequentially decreasing in the C57BL/6J mice (Figures 5G,I), a pattern not reproduced in Gal3***^–^***^/^***^–^*** mice (Figures 5G,J). In C57BL/6J, Notch1 expression extended around 500 μm of distance from the lesion core and just around 250 μm in Gal3***^–^***^/^***^–^*** mice. Additionally, overall Notch1 expression was higher in C57BL/6J mice than Gal3***^–^***^/^***^–^*** mice (Figures 5G,H). These results suggest that in the absence of Gal3, there was an incomplete activation response and low Notch signaling pathway activation after brain injury.

## Discussion

Reactive, mature cortical astrocytes are the first line of response to a brain injury. These cells comprise a large heterogeneous population of glial cells, which display a set of dynamical and complex changes at the molecular, biochemical, and cellular levels. The combination of those changes allows astrocytes to generate a reparative response to cell death and tissue dysfunction. Here we successfully evaluated the changes generated after a TBI at the cellular and molecular level, with the *in vitro* model of astrocyte reactivation, and at the tissular level, using a mice model of TBI.

Given the complexity of astrocyte reactivity, we focused on determining isolated cortical astrocytes response to a mechanical lesion. *In vitro* culture systems for accessing astroglial biology have been used for decades. In face of astrocyte cellular complexity, *in vitro* cultures benefit from offering a simplified microenvironment and a way of studying astrocyte behavior and molecular signaling without the interference of other cell types. On the other hand, it should be mentioned that two-dimensional culture systems for astrocytes limit their morphological complexity and promote an undesired baseline reactivity (Zamanian et al., 2012; Pekny and Pekna, 2014). Noteworthy, protoplasmic astrocytes from the healthy cortex do not express GFAP, but isolated cortical astrocytes display a basal expression of GFAP *in vitro*. However, astrocytes acquire a much higher degree of reactivity upon scratch-activation and can be used as a model of astrocyte reactivation response to traumatic injury (Figures 1B, 4D and Supplementary Figure 5). In this work, we took advantage of a simplified *in vitro* scenario to understand the role of a highly conserved intercellular signaling pathway, Notch1, and a multifunctional lectin binding protein (Gal3) in modulating astrocyte reactivation to trauma. Next, we based our findings on the *in vivo* model of TBI, which offers a higher level of cellular complexity.

First, we show that at 3 days post lesion (3 dpl), Notch1 intracellular domain (NICD1) localizes in the nucleus (Figures 1C,D) and colocalizes with Jagged1 ligand in astrocytes located at the border of the *in vitro* lesion (Supplementary Figure 3). We also observed upregulation of the *Notch1* receptor, *Delta-like1*/*Jagged1* ligands, *Hes5* target gene, and downregulation of the proneural gene factor *Mash1* at the mRNA level in reactive astrocytes (Figures 4E,F), which complements our immunostaining results and strongly indicates Notch1-Hes5 signaling activation upon astrocyte reactivation *in vitro*. Notch signaling is essential during neurodevelopment, adult neurogenesis, and has been implicated in astrocyte reactivity. Previous studies described Notch1 signaling activation in astrocytes after brain injury *in vitro* and *in vivo*. In one study, the number of proliferating astrocytes decreased after treatment with a specific inhibitor of y-secretase, the enzyme responsible for Notch cleavage and release of NICD, in the peri-infarct area of mice submitted to a model of stroke (Shimada et al., 2011). Also, previous studies have shown Jagged1 localization in endosomes (Heng et al., 2020) and its intracellular fragment in the nucleus (LaVoie and Selkoe, 2003). Accordingly, other study demonstrated Jagged1 upregulation on the ischemic ipsilateral side of mice brain, where Notch1/Jagged1 signaling influenced indirectly reactive astrocyte proliferation through induced expression of endothelin receptor type B (LeComte et al., 2015). A later study in a rat model of intracerebral hemorrhage, in which the specific inhibitor of the y-secretase enzyme DAPT was used, showed that Notch1 signaling was upregulated. DAPT blockade of y-secretase suppressed astrocyte proliferation and GFAP expression 14 days post lesion and improved neurological signs (Zhong et al., 2018). Finally, *in vitro* reactive astrocytes showed increased NICD 12 h after being submitted to hypoxia (Zhang et al., 2015).

Conversely, it was shown in an *in vitro* astrogliosis model that LPS induces reactive astrocyte morphology by Notch signaling inhibition (Acaz-Fonseca et al., 2019). Interestingly, Jagged1 upregulation mediated NICD downregulation in those cells. Astrocyte reactivity was also correlated with Notch signaling downregulation in an entorhinal cortex lesion model in mice (Lebkuechner et al., 2015). Moreover, it was sequentially reported that striatal astrocytes of Rbpj deleted transgenic mice lacked nuclear NICD protein 2 weeks after stroke. The same study showed that NICD negative striatal astrocytes generated DCX/Ascl1 neuroblasts from 2 to 7 weeks after stroke, suggesting that Rbpj deletion alone was sufficient to activate their neurogenic program (Magnusson, 2014; Santopolo et al., 2020). The differences in Notch1 signaling outcome in reactive astrocytes is not surprising, as astrocyte reactivation is heterogeneous and dependent upon injury type and severity (Burda and Sofroniew, 2014).

In our scratch-induced astrocyte activation model, we also observed Gal3 overexpression (Figure 2), a finding previously reported in inflammation and injury. Gal3 was increased in white matter reactive astrocytes in a stab wound injury model in adult mouse cerebral cortex, and that was later correlated with reactive astrocyte proliferation *in vitro* (Sirko et al., 2015). Upregulation of Gal3 was also described in cells of the striatum and subventricular zone of patients with perinatal hypoxia/ischaemia (Al-Dalahmah et al., 2020). Furthermore, here we show a GFAP^+^/Gal3^+^ cell population in control and reactive astrocytes, and additionally, that Gal3 is preferentially located intracellularly and in a vesicular pattern in reactive astrocytes (Figures 2, 3). It is important to mention that Gal3 vesicular pattern is correlated to its role in phagocytosis, endocytosis, and lysosome repair (Rotshenker, 2009; Lakshminarayan et al., 2014; Jia et al., 2020). Gal3 is released by activated microglia and acts as a phagocytosis ligand, opsonizing apoptotic cells, myelin, and debris for phagocytosis via Mer tyrosine kinase receptor, which is expressed by microglia and macrophages (Venkatesan et al., 2010; Caberoy et al., 2012; Nomura et al., 2017; Puigdellívol et al., 2020). Interestingly, optic nerve head astrocytes constitutively display a phagocytic phenotype, by internalizing axonal evulsions and upregulating Gal3 upon injury (Nguyen et al., 2011). In any case, there is no evidence, to our knowledge, that Gal3 upregulation in cortical reactive astrocytes drives phagocytic activity. Moreover, another Gal3 role is to mediate clathrin-independent endocytosis, through binding with membrane glycosphingolipids and glycosylated proteins (Lakshminarayan et al., 2014; Stanley, 2014). Gal3 interaction with surface glycoproteins induce membrane deformation and clathrin independent carrier (CLIC) formation. Upon internalization, Gal3 can modulate intracellular signaling pathways involved in apoptosis, cell migration, proliferation, and angiogenesis (Rabinovich et al., 2007; Boscher et al., 2011; Elola et al., 2015). Lakshminarayan and collaborators reported several Gal3 cell-surface binders in mouse mammary tumor epithelial cells, including Notch2 (Lakshminarayan et al., 2014).

Because Gal3 displayed vesicular pattern distribution similar to NICD1 immunostaining (Figures 3B–D and Supplementary Figure 4), we sought to determine if Gal3 and NICD1 colocalized in reactive astrocytes. Our results revealed strong Gal3/NICD1 colocalization in reactive astrocytes in comparison to control (Figures 3E,F). NICD1/Gal3 interaction was previously described in ovarian cancer stem-cells and was related to stemness maintenance. In this study, the authors showed that Gal3 silencing in SKOV3 ovarian cancer stem line decreased the levels of cleaved NICD1 without changing Notch1 receptor expression. Furthermore, it was found that Gal3 interacted with NICD1 through its carbohydrate recognition domain (CRD) (Kang et al., 2016). Our results point to a possible interaction of Gal3 with NICD1 in reactive astrocytes, but whether this interaction occurs and if it involves Gal3 CRD is subject to future studies.

It is well-known that Gal3 interacts with glycosylated proteins through CRD, forming a dynamic and complex structure at the cell membrane which regulates protein diffusion, compartmentalization, and endocytosis (Johannes et al., 2018). Noteworthy, tumor secreted Gal3 was shown to increase Jagged1 half-life at endothelial cell surface, promoting Notch1/Jagged1 signaling (Nascimento dos Santos et al., 2017). The authors showed that Notch1/Jagged1 signaling activation occurs through Gal3 modulation, and in turn, promoted HUVEC spheroid sprouting in an *in vitro* model of tumor angiogenesis. Gal3 modulatory role in Notch1 signaling was also described in preventing osteoblast (Nakajima et al., 2014) and B cell differentiation (De Oliveira et al., 2018). Although we cannot discard Gal3 interaction with Notch receptor and ligands at the cell surface, our analysis suggests Gal3 is mainly distributed in the intracellular compartment of reactive astrocytes, where it might interact with NICD in the cytoplasm and nucleus (Figures 3E,F). Besides, not all NICD1 positive vesicles were Gal3 positive, indicating that we might be facing two different signaling mechanisms, in which one of them, Gal3 could be facilitating Notch signaling, or triggering a distinct signaling response, as to canonical Notch signaling alone.

Finally, at a functional level, and taking advantage of the Gal3^–/–^ transgenic mice, we examined the overall effects of Gal3 ablation on astrocyte reactive response and Notch signaling activation. Normalized fluorescence analysis revealed that GFAP protein level did not rise after Gal3^–/–^ astrocyte reactivation (Figure 4D). This data is in agreement with our *in vivo* results, in which we show fewer GFAP^+^ cells around the TBI border of Gal3^–/–^ mice (Figure 5F, discussed below). *In vitro*, we observed NICD1 absence in the nucleus of reactive and control astrocytes (Figure 4C). Consistent with that, scratch stimuli did not induce changes in *Notch1* receptor, *Jagged1*, and *Delta-like1* ligands and target genes *Hes1* and *Mash1* gene expression (Figures 4E,F). Hes5 downregulation at Gal3^–/–^ reactive astrocytes further indicates that Notch1 signaling is inactive. The scenario was the opposite in C57BL/6J astrocytes: *Notch1* and *Jagged1* were highly upregulated upon lesion stimuli (Figure 4E). It is known that NICD1 induces the expression of *Jagged1* (Manderfield et al., 2012) leading to a positive biochemical feedback in which both cells have high Notch1 and high Jagged1 levels. This mechanism, named lateral induction, was observed during inner ear (Petrovic et al., 2014) and inner heart development (Manderfield et al., 2012). Of note, *Delta-like1* was also upregulated in reactive astrocytes (Figure 4E), although not to the same level as *Jagged1*. Thus, the functional implication of Jagged vs. Delta-like signaling in reactive astrocytes is yet to be determined. We further investigated and determined *Hes5* as the Notch1 effector gene upregulated in C57Bl/6J reactive astrocytes. Hes1 protein negatively regulates *Mash1* proneural gene expression (Kobayashi and Kageyama, 2014), and although *Hes1* mRNA expression level did not change, *Mash1* was downregulated in reactive astrocytes, which is indicative of Notch signaling activation (Figure 4F).

*In vivo*, the absence of Gal3 generates an incomplete response in reactive astrocytes as evidenced by the decrease in the number of GFAP reactive astrocytes and the morphological changes describing atrophy and loss of function in the TBI model. We assessed astrocyte perimeter as an indirect measure of cell domain and reactive response (Figures 5C,D). Gal3^–/–^ GFAP astrocytes have smaller cell body perimeter when compared to C57BL/6J GFAP astrocytes, indicating a loss of complexity, which at the functional level suggests impaired reactive response to TBI. In addition, we showed a decreased number of GFAP reactive astrocytes around the lesion border in Gal3^–/–^ mice. This phenomenon was also described after stab wound injury in gray matter astrocytes of the cortex of Gal1^–/–^ and Gal3^–/–^ mice; however, the authors showed that it reflected a reduction in *Gfap* expression level and in GFAP^+^ cells rather than in astrocyte number (Sirko et al., 2015). Interestingly, a significantly smaller percentage of total GFAP and Olig2 glial cells was found in the striatum and subventricular zone of electroporated mice with Gal3 knockdown constructs and Gal3fl/fl mice. Conversely, in this same study, Gal3 overexpression in wildtype mice increased the proportion of glial cells. Authors suggest that endogenous Gal3 is necessary for striatal gliogenesis from the subventricular zone (Al-Dalahmah et al., 2020).

Mature astrocytes occupy specific cellular domains, which are respected upon mild astrocyte reactivation. When there is a violation of astrocyte territorial domain, such as in traumatic injury, astrocyte reactivity involves territorial overlap and ultimately glial scar formation (Verkhratsky et al., 2017). It was previously shown that reactive astrocytes from the cerebral cortex do not overlap domains, but rather show hypertrophy after electrical lesion (Wilhelmsson et al., 2006; Pekny et al., 2019). Hypertrophic reactive astrocytes are morphologically thicker in their soma and primary and secondary branches, as a consequence of GFAP and vimentin upregulation (Pekny et al., 2019). In contrast, astrocyte atrophy, characterizing domain loss, was correlated to neurological diseases and aging (Verkhratsky and Parpura, 2016; Verkhratsky et al., 2020).

Moreover, in the border of the lesion of Gal3^–/–^ mice there was a poor Notch signaling activation response (Figure 5G). This result suggests a lack of the lateral induction mechanism, where atrophic astrocytes do not communicate properly, affecting the collective cell-fate decision and function. Gal3 alone or Gal3/NICD1 signaling could be involved in astrocyte morphological dysfunction after TBI. Gal3 loss leads to deficient morphological reactive response, which in turn affects cell-cell communication and, consequently, prevents Notch1 signaling to be propagated to the adjacent tissue. In C57Bl/6J mice, we hypothesize that Notch1/Jagged1 signaling is upregulated in astrocytes in the lesion border and propagate to the periphery by lateral induction. The diminished morphological response in Gal3^–/–^ astrocytes could be a consequence of impaired Notch signaling. We hypothesize that in the absence of Gal3, NICD1 does not signal efficiently, which could lead and/or worsen the morphological defects observed. Noteworthy, we did not address cause and consequence, but the data we presented here suggest that these events are linked and operate proper reactive astrocyte responses to TBI.

## Conclusion

**FIGURE 6:**
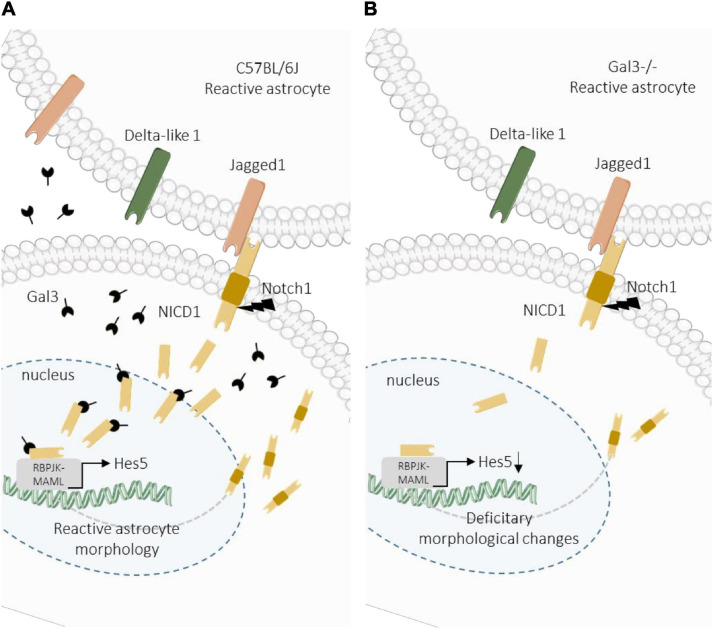
Proposed model for Gal3 modulation of Notch1/Jagged1 signaling in reactive astrocytes. **(A)** After TBI in wild type mice, Notch1/Jagged1 signaling is activated in astrocytes, and NICD1 nuclear translocation and Hes5 transcription activation are Gal3-dependent. Activation of Notch1 signaling promotes astrocyte morphological response to TBI. **(B)** In Gal3^–/–^ mice, after TBI Notch1 signaling is activated at the lesion core, possibly through Jagged1, however activation is less intense compared to wild type mice. We hypothesize that Gal3 modulates NICD1 signaling, which is necessary for astrocyte activation in response to TBI. In the absence of Gal3, NICD1 signaling is disrupted and astrocyte activation is incomplete.

Efforts have been made to unravel the molecular factors and signaling pathways involved in astrocyte reactivation with the final aim to direct astrocytes response for regenerative medicine. Here we provide new evidence of the response of reactive astrocytes and the participation of Notch signaling pathway and Gal3 in the modulation of this response. Our results indicate that Gal3 is essential for proper activation of the Notch signaling pathway, facilitating the cleavage and nuclear translocation of NICD to the nucleus of reactive cortical astrocytes. Additionally, we hypothesize that the reactive astrocyte response is dependent on Notch1/Jagged1/Hes5 signaling activation during a brain injury (Figure 6).

## Data Availability Statement

The original contributions presented in the study are included in the article/Supplementary Material, further inquiries can be directed to the corresponding author/s.

## Ethics Statement

The animal study was reviewed and approved by the Committee on Ethics in the Use of Animals from Universidade Federal de São Paulo (Comitê de Ética no Uso de Animais; CEUA numbers 7740290318 and 2451111116).

## Author Contributions

TR performed experiments, wrote the original draft, reviewed and edited the final document and figures, and funded by grant foundations. LD-G performed experiments, wrote the original draft, reviewed and edited the final document and figures, and received fellowship from grant foundations. MP conceptualized the study, advised TR and LD-G during execution of experiments, reviewed and edited the final document, and received funding from grant foundations. All authors contributed to the article and approved the submitted version.

## Conflict of Interest

The authors declare that the research was conducted in the absence of any commercial or financial relationships that could be construed as a potential conflict of interest.

## Footnotes

1 https://www.premium.fm.usp.br/

2 http://rsbweb.nih.gov/ij

3 https://www.graphpad.com/scientific-software/prism/

4 https://www.microsoft.com/pt-br/microsoft-365/excel

## Supplementary Material

Supplementary Figure 1 Gal3^–/–^ mice genotyping. Adult male mice (45 days) isogenic, Lgals3^–/–^ (Gal3^–/–^) (Hsu et al., 2000) and wild type (C57BL/6J, Lgals3^+/+^) were genotyped using the “*quick-dirty HotSHOT*” (Truett et al., 2000) method. Tail samples from Gal3^–/–^ (*n* = 3) and wild-type (*n* = 1) animals were incubated with lysing agent (25 mM NaOH and 0.2 mM EDTA) (95°C, 60 min; hold 4–15°C) and neutralized with 75 μl 40 mM TrisHCl. The samples were centrifuged (4,000 rpm, 3 min, −20°C) and analyzed by conventional PCR. Gal3^–/–^ (*n* = 3) and C57BL/6J (*n* = 1). Agarose gel (1.5%) electrophoresis of PCR products. The 300 bp band corresponds to Gal3 in Gal3^–/–^ mice and 490 bp to Gal3^+/+^ in C57BL/6J mice.

Supplementary Figure 2 NICD1 immunostaining pattern in C57Bl/6J reactive astrocytes. Representative confocal Z-stack images of NICD1 immunostaining in astrocytes at 3 days post scratch-reactivation stimuli. NICD1 is found in the nucleus and in cytoplasmic vesicles. Arrowheads indicate astrocytes with nuclear and vesicular NICD1. Arrows indicate astrocytes with nuclear NICD1. Scale bar: 20 μm.

Supplementary Figure 3 NICD1 and Jagged1 colocalize in reactive astrocytes at the border of the *in vitro* lesion. **(A)** Colocalization images for NICD1/Jagged1 reveal strong colocalization at the lesion core compared to the periphery. White dots indicate NICD1/Jagged1 colocalization. **(B)** Representative confocal image of GFAP/NICD1/Jagged1 stained reactive astrocytes. The dashed line represents the two comparative regions, lesion core and periphery. The core region extends 100 μm from the border of the scratch. Scale bar: 50 μm. **(C)** Colocalization coefficient was used for statistical analysis (^∗∗∗^*p* ≤ 0.001; unpaired Student’s *t*-test, *n* = 19 cells at lesion core / 21 cells in periphery; 9 images were analyzed from three culture replicates). Data are mean ± SEM.

Supplementary Figure 4 NICD1 and Gal3 distribution patterns. **(A)** Representative confocal Z-stack images of the four labeling patterns for NICD1 and Gal3 in cortical astrocytes *in vitro*. Reactive astrocytes display NICD1 vesicular pattern, and control astrocytes present NICD1 diffuse pattern distribution. Arrowheads indicate vesicles. Scale bar: 20 μm. **(B)** Schematic representation of NICD1 vesicle distribution patterns in control and reactive astrocytes.

Supplementary Figure 5 GFAP in C57Bl/6J and Gal3^–/–^ astrocytes *in vitro.* Representative confocal images of GFAP immunostaining in C57Bl/6J and Gal3^–/–^ astrocytes used for normalized fluorescence analysis. Dashed lines indicate scratch border. Scale bar: 50 μm.
