# Supplementary figures and images for: Notch1 and Galectin-3 Modulate Cortical Reactive Astrocyte Response After Brain Injury
Source: Front Cell Dev Biol. 2021 Jun 16;9:649854. doi: 10.3389/fcell.2021.649854 (PMC8244823; doi:10.3389/fcell.2021.649854)

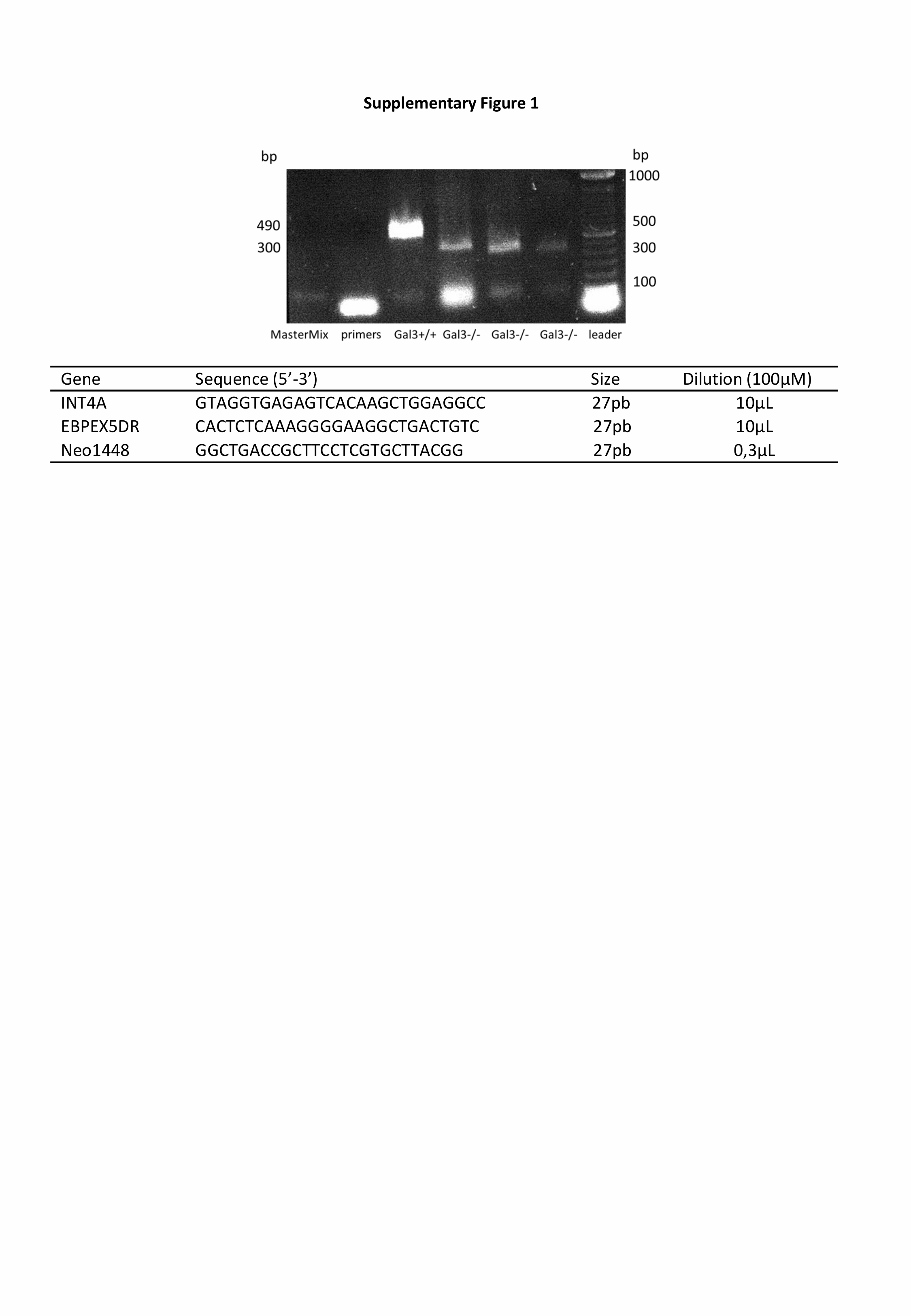

Supplement: Supplementary Figure 1 — Gal3–/– mice genotyping. Adult male mice (45 days) isogenic, Lgals3–/– (Gal3–/–) (Hsu et al., 2000) and wild type (C57BL/6J, Lgals3+/+) were genotyped using the “quick-dirty HotSHOT” (Truett et al., 2000) method. Tail samples from Gal3–/– (n = 3) and wild-type (n = 1) animals were incubated with lysing agent (25 mM NaOH and 0.2 mM EDTA) (95°C, 60 min; hold 4–15°C) and neutralized with 75 μl 40 mM TrisHCl. The samples were centrifuged (4,000 rpm, 3 min, −20°C) and analyzed by conventional PCR. Gal3–/– (n = 3) and C57BL/6J (n = 1). Agarose gel (1.5%) electrophoresis of PCR products. The 300 bp band corresponds to Gal3 in Gal3–/– mice and 490 bp to Gal3+/+ in C57BL/6J mice. [file Image_1.TIF]

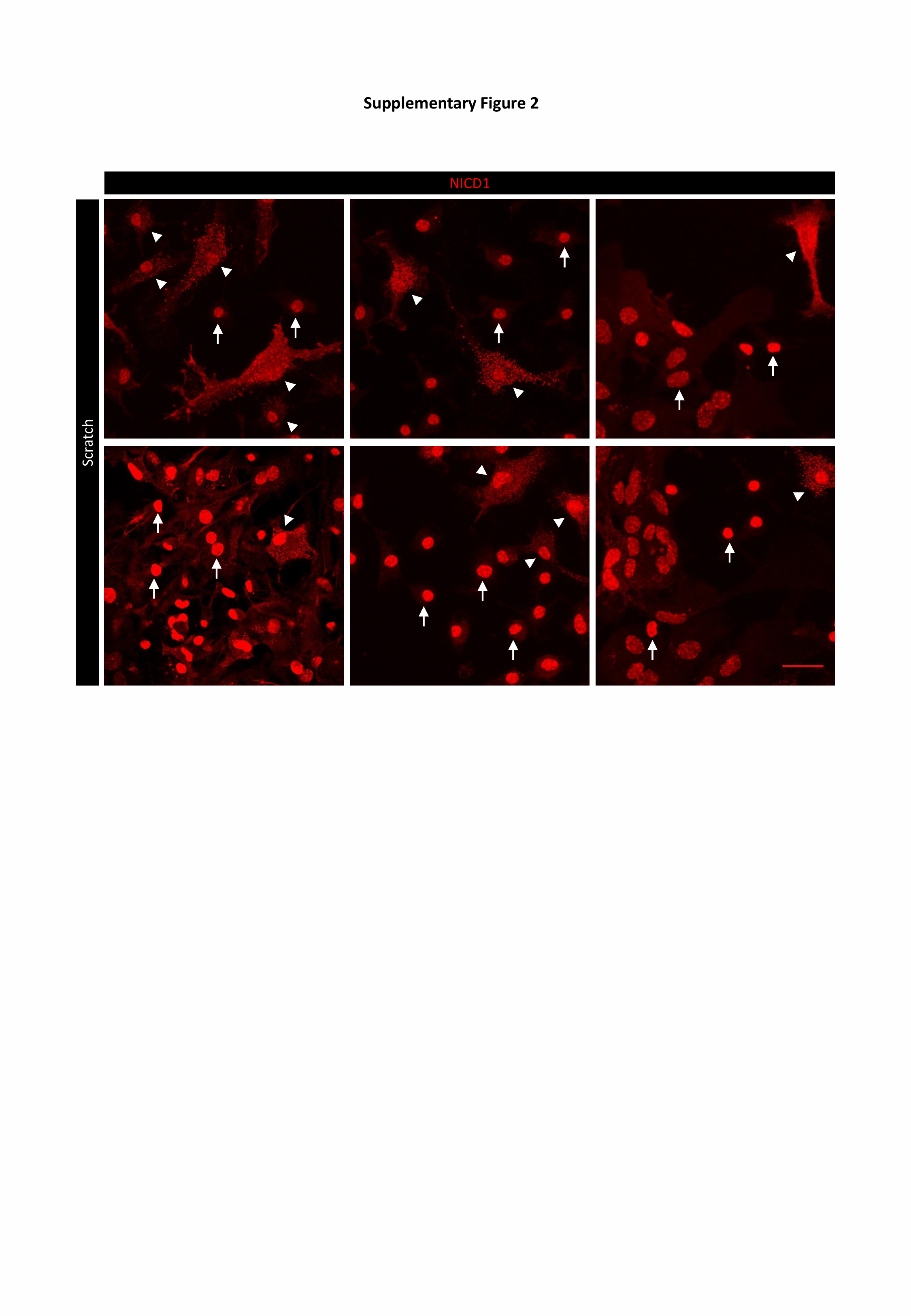

Supplement: Supplementary Figure 2 — NICD1 immunostaining pattern in C57Bl/6J reactive astrocytes. Representative confocal Z-stack images of NICD1 immunostaining in astrocytes at 3 days post scratch-reactivation stimuli. NICD1 is found in the nucleus and in cytoplasmic vesicles. Arrowheads indicate astrocytes with nuclear and vesicular NICD1. Arrows indicate astrocytes with nuclear NICD1. Scale bar: 20 μm. [file Image_2.TIF]

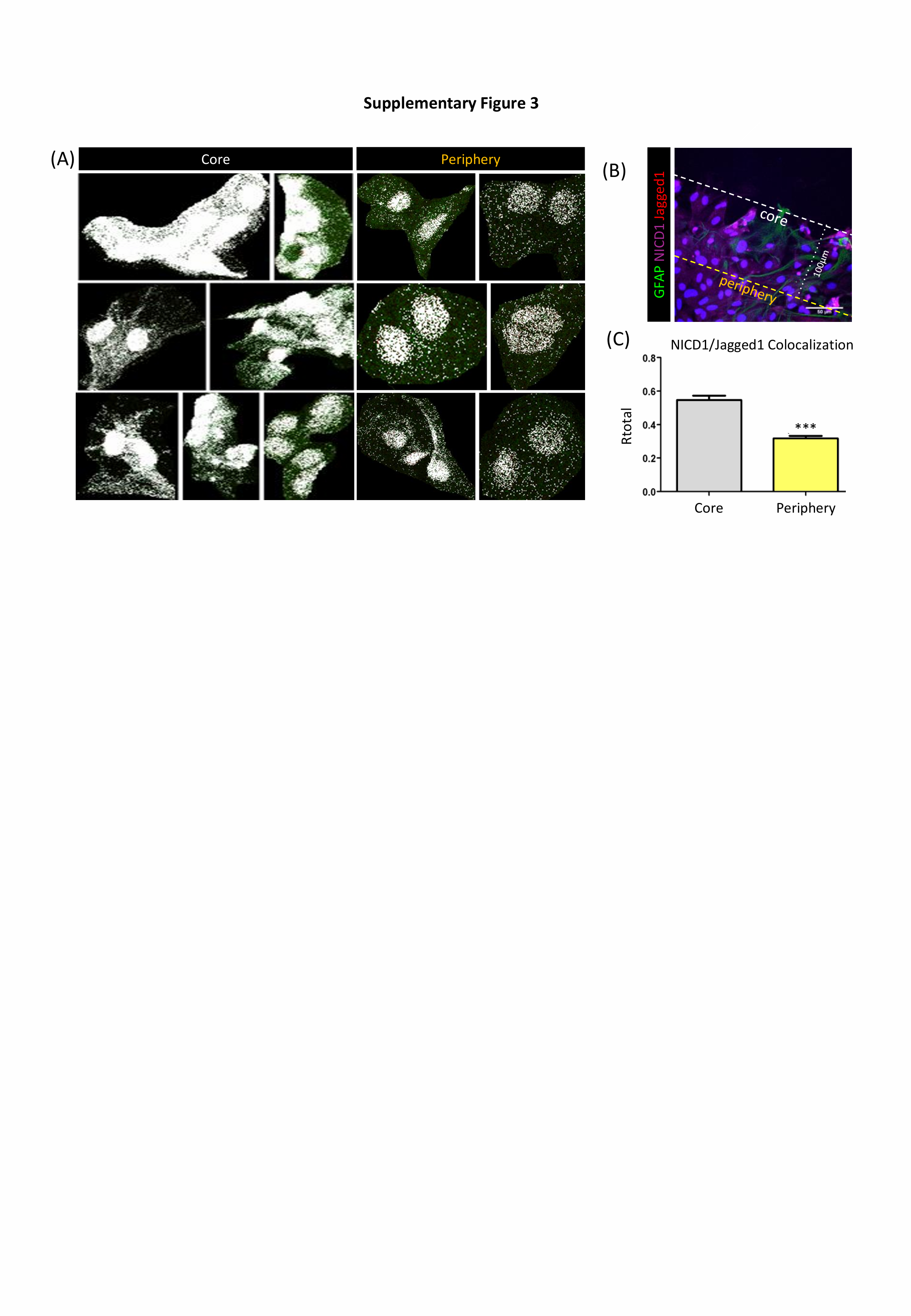

Supplement: Supplementary Figure 3 — NICD1 and Jagged1 colocalize in reactive astrocytes at the border of the in vitro lesion. (A) Colocalization images for NICD1/Jagged1 reveal strong colocalization at the lesion core compared to the periphery. White dots indicate NICD1/Jagged1 colocalization. (B) Representative confocal image of GFAP/NICD1/Jagged1 stained reactive astrocytes. The dashed line represents the two comparative regions, lesion core and periphery. The core region extends 100 μm from the border of the scratch. Scale bar: 50 μm. (C) Colocalization coefficient was used for statistical analysis (∗∗∗p ≤ 0.001; unpaired Student’s t-test, n = 19 cells at lesion core / 21 cells in periphery; 9 images were analyzed from three culture replicates). Data are mean ± SEM. [file Image_3.TIF]

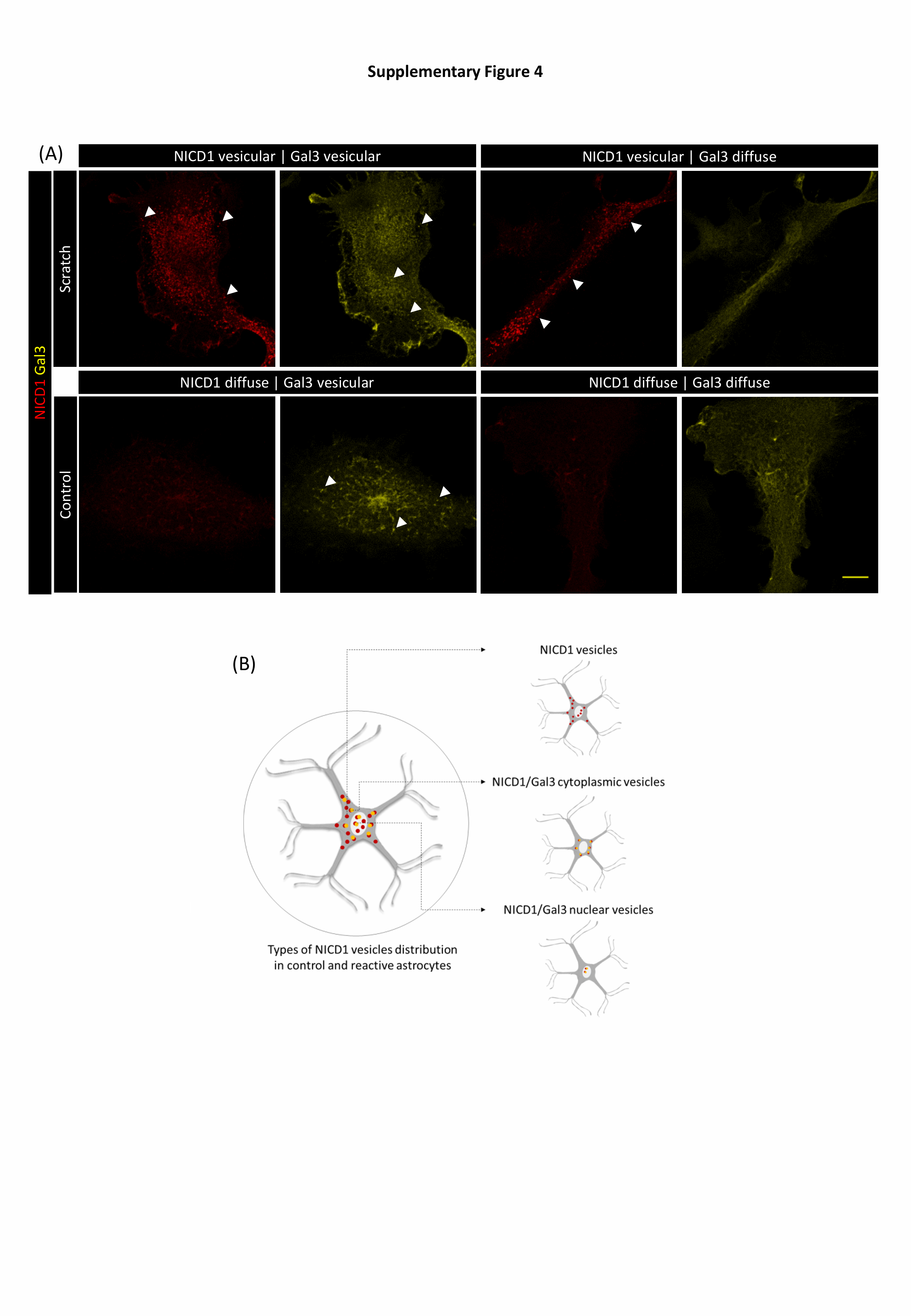

Supplement: Supplementary Figure 4 — NICD1 and Gal3 distribution patterns. (A) Representative confocal Z-stack images of the four labeling patterns for NICD1 and Gal3 in cortical astrocytes in vitro. Reactive astrocytes display NICD1 vesicular pattern, and control astrocytes present NICD1 diffuse pattern distribution. Arrowheads indicate vesicles. Scale bar: 20 μm. (B) Schematic representation of NICD1 vesicle distribution patterns in control and reactive astrocytes. [file Image_4.TIF]

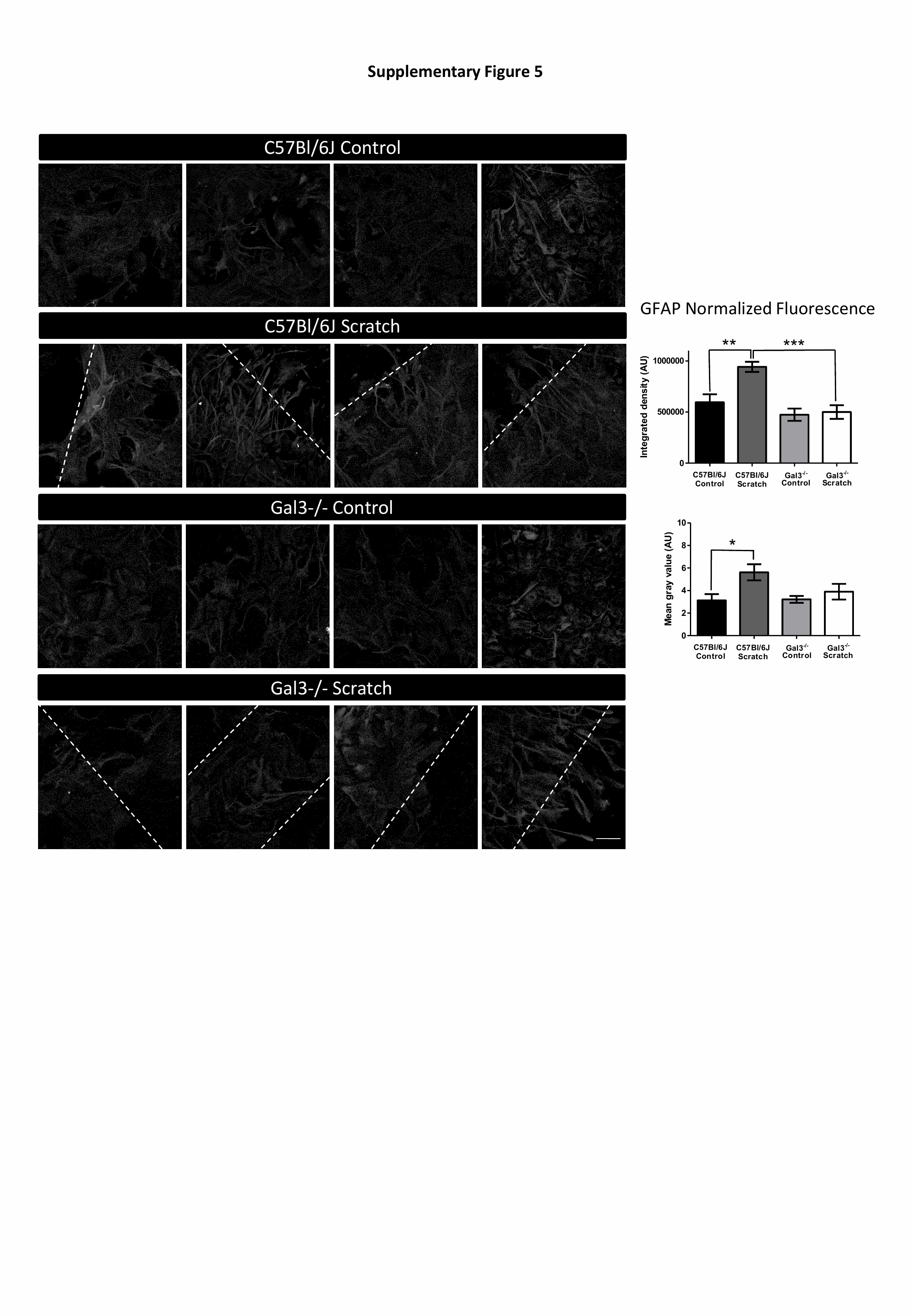

Supplement: Supplementary Figure 5 — GFAP in C57Bl/6J and Gal3–/– astrocytes in vitro. Representative confocal images of GFAP immunostaining in C57Bl/6J and Gal3–/– astrocytes used for normalized fluorescence analysis. Dashed lines indicate scratch border. Scale bar: 50 μm. [file Image_5.TIF]
